# Exposure assessment of radon in the drinking water supplies: a descriptive study in Palestine

**DOI:** 10.1186/1756-0500-5-29

**Published:** 2012-01-13

**Authors:** Hamzeh Al Zabadi, Samar Musmar, Shaza Issa, Nidal Dwaikat, Ghassan Saffarini

**Affiliations:** 1 Public Health and Community Medicine Department, Faculty of Medicine and Health Sciences, An-Najah National University, Nablus, Palestine; 2 Faculty of Medicine and Health Sciences, An-Najah National University, Nablus, Palestine; 3 Radiation Physics Laboratory, An-Najah National University, Nablus, Palestine

## Abstract

**Background:**

Radon gas is considered as a main risk factor for lung cancer and found naturally in rock, soil, and water. The objective of this study was to determine the radon level in the drinking water sources in Nablus city in order to set up a sound policy on water management in Palestine.

**Methods:**

This was a descriptive study carried out in two phases with a random sampling technique in the second phase. Primarily, samples were taken from 4 wells and 5 springs that supplied Nablus city residents. For each source, 3 samples were taken and each was analyzed in 4 cycles by RAD 7 device manufactured by Durridge Company. Secondly, from the seven regions of the Nablus city, three samples were taken from the residential tap water of each region. Regarding the old city, ten samples were taken. Finally, the mean radon concentration value for each source was calculated.

**Results:**

The mean (range) concentration of radon in the main sources were 6.9 (1.5-23.4) Becquerel/liter (Bq/L). Separately, springs and wells' means were 4.6 Bq/L and 9.5 Bq/L; respectively. For the residential tap water in the 7 regions, the results of the mean (range) concentration values were found to be 1.0 (0.9-1.3) Bq/L. For the old city, the mean (range) concentration values were 2.3 (0.9-3.9) Bq/L.

**Conclusions:**

Except for Al-Badan well, radon concentrations in the wells and springs were below the United State Environmental Protection Agency maximum contaminated level (U.S EPA MCL). The level was much lower for tap water. Although the concentration of radon in the tap water of old city were below the MCL, it was higher than other regions in the city. Preventive measures and population awareness on radon's exposure are recommended.

## Background

Radon is a colorless, odorless and tasteless gas. It is a chemically and biologically inert noble gas with a heavily neutron-rich nucleus that makes it a radioactive element [1]. It has three main natural isotopes; radon-222 (Rn-222); radon-220 (Rn-220 also known as thoron); and radon-219 (Rn-219) [2]. Rocky, mountainous regions and phosphate rich soil regions and water, all over the world, tend to have varying amounts of Rn-222 [3,4]. Radon is unstable and breaks down into radon progeny emitting highly ionizing alpha radiation which is very harmful to humans when they are inhaled or swallowed [5].

Radon comes mainly from the soil underneath the building. However, the primary routes of potential human exposure to radon are inhalation radon gas and ingestion of water-dissolved radon [4]. Radon in the groundwater or building materials enters the working and living spaces and disintegrates into its decay products. Although high concentrations of radon in groundwater may contribute to radon exposure through ingestion, the exposure risk through inhalation of radon released from water is usually more significant. Some radon and its progeny in drinking water might be ingested and reach the stomach and intestine. However, inhaled radon into lungs could be readily breathed out through pulmonary circulation [1].

When radon gas is inhaled, the highly-ionizing alpha particles emitted by deposited short-lived decay products of radon Polonium-218 (Po-218) and Polonium-214 (Po-214) can interact with the biological tissue in the lungs leading to DNA damage that is considered as an important step in the carcinogenesis process [6,7]. In the USA, studies showed that radon in homes caused 21,100 lung cancer deaths per year making it the second leading cause of lung cancer deaths [7]. In UK, it is estimated to be responsible for about 1,100 deaths per year [8].

Table 1 below shows some international studies that were carried out on different water sources and their measured radon concentration levels. A further study showed that the groundwater sources (springs and wells) were generally more enriched in Rn-222 than surface waters (rivers and streams) [9]. In the Middle East, Jordan for example, a neighboring country to Palestine, a study was performed on the radon level in water [10]. The concentration of Rn-222 ranged from 3.3 to 10.7 Bq/L (Becquerel/liter) in cold spring water, from 3.2 to 5.5 Bq/L in hot spring water, from 3.1 to 5.7 Bq/L in well water, from 2.5 to 4.7 Bq/L in drinking water and from 4.3 to 6.3 Bq/L in the sea water. The study concluded that these measurement levels were within the usual standard limits of radon. In another neighboring country, Lebanon, a study on water sources was performed and found that the water dissolved radon concentrations ranged from a low of 0.91 Bq/L in a coastal well source to a high of 49.6 Bq/L in a spring source in a mountainous region. Of the 20 sites sampled, only five had radon levels above 11 Bq/L (the U.S EPA maximum contaminant level) [11] and these mostly occurred in areas adjacent to well-known geological fault zones. In general, the concentrations found in the previous mentioned studies were all below the 100 and 146 Bq/L level proposed by the European Union [12] and the United States alternative maximum contaminant level (AMAL) [11]; respectively.

**Table 1:** Some international studies that were carried out on different water sources and their measured radon concentration levels

Country	Water source	Radon concentration	Reference	Notes
United Kingdom	Tap water	1-2 Bq/L (range)	[9]	The mean radon level was below 11 Bq/L which is the safe level approved by the United States Environmental Protection Agency (U.S EPA) [10].

Finland	Drill Wells	130 Bq/L (mean)	[11]	Aimed to find if there is a relationship between radon from ingestion of water and stomach cancers. The risk for stomach cancer from radon was 0.69 (not significant).

Italy	Ground water/Volcanic area	1.8-52.7 Bq/L (range)	[12]	About 40% of the samples (n = 119) exceeded the maximum contaminant level of 11 Bq/L proposed by the U.S EPA.

In Palestine, most studies concerned with the measured indoor radon exposure concentration. For instance, a study was designed to detect the indoor radon concentration in old Nablus city houses [10]. In that study, radon indoor levels were in the normal range according to USA radon safe level [11] but they exceeded the levels found in some Arab countries as Saudi Arabia, for example. Although Palestine has a Mediterranean climate and the people usually use natural ventilation in summer through windows and doors that could lead to relatively low indoor radon concentration in summer, the high indoor radon concentration in that specific region in Palestine (old city) could be explained most probably due to the overcrowding of the old city houses and therefore poor ventilation. In Israel, that share the same environmental conditions with Palestine, a survey was carried out between 1998 and 2003 to determine the indoor radon level [13] showed that there were 6 prone areas for radon according to the ICRP (the International Commission on Radiological Protection) definition. The study concluded that radon measurements and mitigation steps should be taken in public and private dwellings in these areas.

Since radon is a health hazard and a risk factor for some types of cancers including lung cancer [7], many studies have been conducted worldwide to determine its concentration in different environmental media in order to reduce its adverse effects on the human health [9,14-16]. To our knowledge, no study was carried out regarding radon assessment in the drinking water supplies in Palestine. Since people are in a daily-contact with the drinking water, it was reasonable to design a study in order to investigate the radon levels in the drinking water in Palestine to set up a sound policy on water management. Nablus city is one of the most populated cities in the West Bank [17] and therefore it was chosen as the study setting. The results of this study will call upon further analytical studies in the West Bank cities on radon concentration in the drinking water. They may also highlight intervention programs and strategies that could help the decision-makers in the better assessment and evaluation of the safety of the drinking water.

## Methods

### Study design

This was a descriptive study performed on two phases. In the first phase we covered all the wells and springs in Nablus city. In the second phase we selected a randomized sample that represents the residential tap water of the city.

### The study area

Nablus city is located in the north of the West Bank and lies on two high mountains. Nablus citizens are provided by water from the municipality water net that drives the water from the main sources to the customers. Those citizens do not use other sources of water like a well in the house garden that is usually filled from rain or tanks as other cities and villages in the West Bank. Nablus water comes from artesian wells and natural springs. There are 6 wells, 4 main springs and 2 other small springs. Water from these wells and springs is pumped to collecting reservoirs and then to the houses and buildings' reservoirs and finally to the residential taps. There are 22 reservoirs, each reservoir received its water from different wells and springs so water arrived to one house does not come from a single main source. The municipality divides Nablus into 7 regions; each region is supplied mainly by a specific reservoir but this also may be varied according to the availability of water. This means, if there is no enough water in the reservoir; water will be pumped from other reservoir to that reservoir then pumped to the houses. There is an 8th region (old city of Nablus) which is supplied only by one spring called Qaryoon spring.

### Sampling framework and data collection

In this study, samples were collected from the main sources (i.e. wells and springs), and from the residential tap water (selected randomly) in the eight regions of Nablus. The main wells are: Deir Sharaf and Sabstia wells in the northwest region, Badan and Faraa wells in the northeast region and Audala and Rujeeb wells in the southeast region. The springs are: Qaryoon, Ein Beit El Ma, Ein Dafna, Ras Al-Ein, Ein Al-Asal and New reservoir (commercial centre). While the regions or zones of Nablus are: Ein Dafna Zone, Northern Zone, Southern Zone, El Sumara zone, Ein Beit el Ma Zone, Ras Al Ein Zone, reservoir zone and the Old city.

Samples were collected in glass bottles (250 ml each). Three samples were taken from each main source. From each region we took 3 samples from the residential tap water while 10 samples were taken from the old city. Regarding the main sources, we were allowed to collect samples only from 9 sources as 3 of them were closed and were not pumping water at the time we conducted the study. The closed sources were Deir Sharaf well, Rujib well and new reservoir spring.

### Experimental setup and measurement procedure

Research project was approved by the research committee at An-Najah National University faculty of medicine after the approval of the Institutional Review Board (IRB). Nablus municipality approval was also obtained. The researcher then received training in using the DURRIDGE RAD7 H_2_O device following the manual instructions [18]. Therefore, we used RAD water device, an accessory to the RAD7 device manufactured by DURRIDGE Company [18]. It was calibrated on 31 June 2010 and the next calibration time should be done in 31 June 2011.

**Figure 1:**
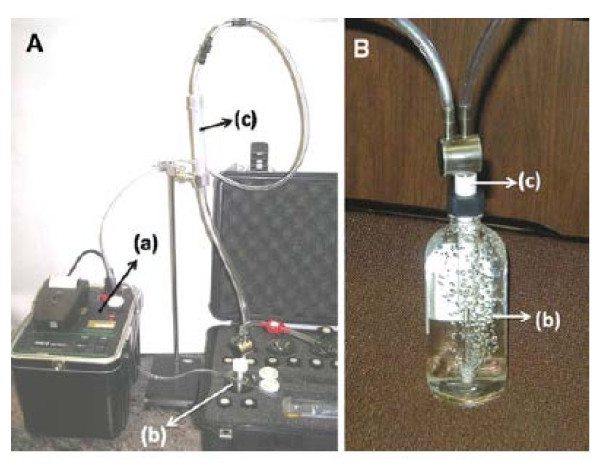
**Schematic presentations of radon-in-air monitor RAD-7**. Adapted from reference [18] with permission.

Briefly, this device offers an accurate measurement, faster reading, it is portable and eliminates the need for noxious chemicals. The schematic diagram of this device is presented in Figure 1 below. Using RAD H2O technique employs closed loop concept, consisting of three components; (a) the RAD7 or radon monitor, on the left, (b) the water vial with aerator, in the case near the front, and (c) the tube of desiccant, supported by the retort stand above as marked in the figure.

The RAD-H20 method employs a closed loop aeration scheme whereby the air volume and water volume are constant and independent of the flow rate. The air re-circulates through the water and continuously extracts the radon until a state of equilibrium develops. The RAD-H20 system reaches this state of equilibrium within about 5 min, after which no more radon can be extracted from the water. The operation of this device is based on the following principle; (1) radon is expelled from a water sample by using a bubbling kit, (2) expelled radon enters a hemisphere chamber by air circulation, (3) polonium decayed from radon is collected onto a silicon solid-state detector by an electric field and (4) radon concentration is estimated from the count rate of polonium [18].

On the RAD7, one among the two available protocols (i.e., Wat-40 and Wat-250) will be selected depending on the size of vial (40 or 250 mL) that is being used for water sampling (here we used Wat-250 and sample size of 250 mL). This also decides the extraction efficiency or percentage of radon removed from the water to the air loop. For our used protocol of Wat-250, the extraction efficiency was usually very high, typically 95% for a 250 mL sample vial [18].

The 250 mL sample bottle was connected to the RAD-7 and the internal air pump of the radon-monitor was used for re-circulating a closed air-loop through the water sample, purging radon from the water into the air-loop. The air is re-circulated through the water continuously to extract the radon until RAD-H2O system reaches a state of equilibrium within about 5 min, after which no more radon can be extracted from the water. After reaching equilibrium between water, air, and radon progeny attached to the passivity implanted planar silicon detector, the radon activity concentration measured in the air loop was used for calculating the initial radon-in-water concentration of the respective sample. The RAD-7 allows determination of radon-in-air activity concentrations by detecting the alpha decaying radon progeny Po-218 and Po-214 using passivity implanted planar silicon detector. The radon monitor (RAD-7) uses a high electric field above a silicon semi-conductor detected at ground potential to attract the positively charged polonium daughters (Po-218 and Po-214) which are counted as a measure of radon-222 concentration in air.

The pump runs for 5 min, aerating the sample and delivering the radon to the RAD7. The system will wait a further 5 min and then it starts counting. During the 5 min of aeration, more than 95% of the available radon is removed from the water and the components automatically perform everything required to determine the radon concentration in the water. After 5 min, it prints out a short-form report.

The same thing is repeated again for 5 min later, and for two more 5-min periods after that. Thus, radon gas is collected through the energy specific windows which eliminate interference and maintain very low backgrounds and later counted for the radon concentration. Radon-222 activities are then expressed with uncertainty down to under ± 5%. At the end of the run (30 min after the start), the RAD7 prints out a summary, showing the average radon readings from the four cycles, counted a bar chart of the four readings, and a cumulative spectrum.

The RAD H20 enables the measurement of radon in water over a concentration range between 30 and 10^5 ^pCi/L (pico-Curie/liter). The lower limit of detection was less than 10 pCi/L [18]. The exact value of the extraction efficiency depends somewhat on ambient temperature, but it is almost always well above 90%. Furthermore, the temperature effect on accuracy is usually noticeable with the 250 mL vial at only very low or high temperatures. The RAD-H20 system has been calibrated for a sample analysis temperature of 20 C°. In our study, the mean ± standard deviation (M ± SD) temperature value for the wells' and springs' samples was 23.6 ± 0.74 and for the residential tap water samples was 20.0 ± 1.15. Therefore, a very limited or no effect of temperature was seen on the results.

The RAD7 calculates the sample water concentration by multiplying the air loop concentration by a fixed conversion coefficient that depends on the sample size. This conversion coefficient has been derived from the volume of the air loop, the volume of the sample, and the equilibrium radon distribution coefficient at room temperature. For the 250 mL sample volume, the conversion coefficient was around 4 [18]. In the analysis, we converted the picocurie (pCi/L) into Becquerel (Bq/L) unit using the formula that 1 pCi = 0.037 Bq.

Samples were taken in specific bottles designed for the RAD device and provided by the manufacturer. The collections of the samples and their analysis (for springs and wells) were done between 27th of November 2010 and 4th of December 2010. Tap water sampling and analysis from different regions of Nablus area were done between 7th of December 2010 and 20th of December 2010.

### Sample analysis

To ensure the quality control and reliability of the sampling and measurement methods, each sample was analyzed in 4 cycles. The mean for these 4 cycles was then calculated in regard to the wells and springs [see Additional file 1: Wells and springs original data set in Bq]. Regarding the residential tap water, we took 3 samples from the houses receiving water from each region using the simple randomization. Each sample was analyzed in 4 cycles where we calculated the mean of these 4 readings and finally we calculated the mean for the 3 samples' means [see Additional file 2: Residential tap water original data set in Bq; see Additional file 3: Old city original data set in Bq].

Concentrations was measured by pCi/L unit as provided by the manufacturer then converted to Bq/L for easier comparison with the literatures. Analysis took place at the radon research laboratory at An Najah National University. All samples were analyzed within 3 days of collection because radon half life is 3.8 days [19]. The relative standard deviations of all the 4 cycles analyzed were within the 10% for their corresponding mean. The SPSS (statistical package for social sciences) software 16 was used for analysis [20].

## Results

### Radon levels in the springs and wells

**Figure 2:**
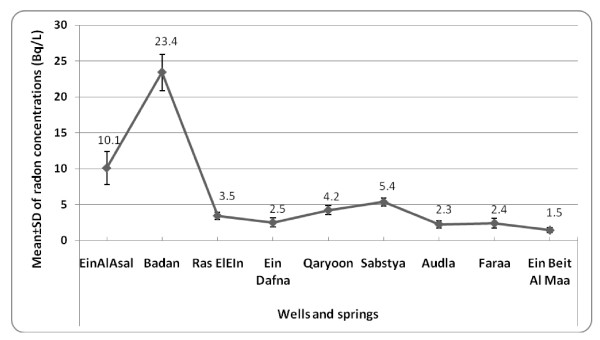
**Radon concentrations in Nablus city' springs and wells in Becquerel/L (Bq/L)**. The bars are related to the means ± SD values of radon concentrations.

The results of radon concentration levels in springs and wells are shown in Figure 2. As presented, radon concentrations ranged from 1.5 to 23.4 Bq/L with a mean of 6.9 Bq/L. In springs alone, the mean (range) values were 4.6 (1.5 to 9.9) Bq/L. In wells however, radon concentration ranged from 2.9 to 23.4 Bq/L with a mean of 9.5 Bq/L. The highest radon concentration was observed in Al-Badan well (mean ± SD; 23.4 ± 0.2 Bq/L).

### Radon levels in the residential tap water

**Figure 3:**
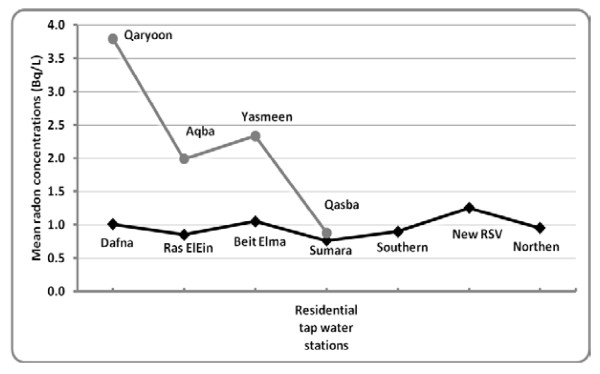
**Radon concentrations in the residential tap water of the seven main regions in Nablus city () and in the four different harats (sub-regions) of the old city region () in Becquerel/L (Bq/L)**.

Figure 3, shows the radon concentration levels in the residential tap water in the 7 regions of the Nablus city. The concentration range was from 0.9 to 1.3 Bq/L with a mean of 1.0 Bq/L. We also presented the old city radon concentration levels on the same figure (Figure 3). The concentration range for old city was from 0.9 Bq/L to 3.8 Bq/L with a mean of 2.3 Bq/L.

In table 2, however, we summarized the minimum, maximum and mean values of radon concentrations in the different assessed sites (springs, wells and tap water) of Nablus city water.

**Table 2:** Maximum, minimum and mean values of radon concentrations in Becquerel/L (Bq/L) in the different assessed sites of Nablus city water

Water assessment site	Minimum value	Maximum value	Mean value
**Springs**	1.5	9.9	4.6

**Wells**	2.9	23.4	9.5

**Tap water**			

-Seven regions	0.9	1.3	1

-Old city	0.9	3.9	2.3

## Discussion

### Radon concentration in springs and wells

The main study finding points that all the readings for wells and springs were lower than the U.S maximum contaminated level (MCL) of 11.1 Bq/L [15] except for one source (Al-Badan). Although Al-Badan well radon concentration level was higher than the U.S MCL, it remained lower than both the Union reference level set at 100 Bq/L [21], and the currently debated U.S. alternative maximum contaminated Level set at 146 Bq/L as an upper limit for drinking water in the United States. These generally low concentration levels of radon in wells and springs could be explained from the geological context of the surrounding rocks. Indeed, according to the information provided by Nablus municipality, the deepest layer in the studied areas was dolomite, followed by limestone and then chalky stones near the surface. This geological structure was similar for almost all the studied wells and springs. Indeed, uranium and radon are found in small amount in all the types of rocks. However, some types of rocks have more concentration of uranium and radon than others. These include light-colored volcanic rocks, granites, dark shales, sedimentary rocks that contain phosphate, and metamorphic rocks derived from these rocks. Dolomite and limestone are sedimentary stones but they are composed of calcium not phosphate which may explain the low concentration values obtained in our study (Dr. Hafez Shaheen, water department in Nablus Municipality, personal communication, June 20, 2011).

However, according to the municipality of Nablus, Al-Badan well was closed most of the time within a month prior to the day of sample collection which might explain the relatively high radon concentration in this well. Another possible explanation is that this well is the deepest among others. Usually radon concentration increases as the depth in earth increases [1]. Furthermore and from the geological and geographical point of view, Al-Badan is located closer to the Dead Sea which is considered as a fault zone. Some studies, however, found a relation between high radon concentration in water, soil and air and seismic activities and earth quake. Indeed, the exhalation rate was found to increase near the fault [11,22,23].

Because water pumped to most of the residential areas in Nablus city is mixed in the collecting reservoirs and therefore, there was no specific main area supplied from Al-Badan well, the effect of high concentration level of radon in this well on the residential areas covered in this work was not feasible to be studied. However, according to the results that we had from the tap water, we could conclude that there was no increase in the exposure for the general population from Al-Badan well if the municipality continues to supply the water in the current mixing source way.

As there is no absolute safe value of radiation from radon on general public [15], each country had its suggested target safe limit. Although there are few studies on radon level in the outdoor and indoor in Palestine [14,24,25], no water radon level reference has been established and therefore, there has been no specific safe limit value for radon until now in Palestine. Even in the neighbor countries, no standard safe level has been developed and they still depend on the U.S or European standard safe levels. In comparison with radon concentration in Jordan, the mean level for cold spring in Irbid ground water basin was found to be 5.4 Bq/L. This concentration level is very close to that found in our study (springs, 4.6 Bq/L) [24]. In Lebanon, the mean was found to be around 11.4 Bq/L which is within our range values. Another study in Saudi Arabia [26] found that the concentration level ranged from 0.81 to 3.56 Bq/L. Our findings regarding radon concentrations in spring and wells are in accordance with the previous studies.

### Radon concentration in the residential tap water

Our findings regarding the residential tap water showed that radon concentration levels were less than the main sources. Several factors might explain the findings: radon decay and radon aeration, mixture of water from different sources before pumping, and the travel distance and time could all play a role [21]. As shown in Figure 3, radon concentration levels between the different regions (except for the old city region) are almost around each other. However, the old city concentrations (mainly Harat Qaryoon) were generally higher than the other regions. This variation may be explained by that the old city is supplied only by one spring (Qaryoon spring) which is located in the old city (Qaryoon sub-region). Therefore, short and close travel distance and consequently lower decay of radon might explain the higher concentrations observed in the old city in general and Harat Qaryoon in specific.

Compared to other countries, the range for tap water in Jordan was found to be 2.5-4.7 Bq/L. This was slightly higher than our study values but near the range of Harat Qaryoon. Our range was also very close to a Saudi Arabia range values of 0.92-2.12 Bq/L [27] and much similar to that in United Kingdom (0-2 Bq/L) [14].

To our concern, the highest level was in Harat Qaryoon, this might reflect a potential high concentration level to the population on long-term exposures. Moreover, the houses in Harat Qaryoon were crowded with poor ventilation and this could play a further role in the potential increased exposure levels.

## Conclusions

Compared with the international references, our findings showed that there was no increase in the exposure of radon in the different drinking water sources in Nablus city. Further studies with larger sample size and different sampling methods might be required to better highlight the exposure levels and risk on the population. As Al-Badan well had a high concentration of radon, further investigations by repeating the measurement or using other method may be needed. We also recommend investigating the radon concentrations in the water sources of other regions in the West bank and Gaza. However, community awareness and education are necessary mainly regarding the ventilation techniques. Attract the attention of the municipality of Nablus city to take preventive measures that could reduce the exposure level of radon in the old city water's supply is also recommended.

## Availability of supporting data

The data sets supporting the results of this article are included within the article (and its additional file (s)).

## Abbreviations

USA: United States of America; UK: United Kingdom; U.S EPA: United States Environmental Protection Agency; Bq/L: Becquerel/liter; MCL: Maximum Contaminated Level; pCi: pico-Curie.

## Competing interests

The authors declare that they have no competing interests.

## Authors' contributions

HA drafted the manuscript. SM and SI participated in drafting the manuscript. HA, SM and SI performed the statistical analysis and participated in the coordination of the study protocol and study design. ND and GS participated in the study design, protocol and in experimental analysis of samples and provided further contributions and suggestions. All authors read and approved the final manuscript.

## Supplementary Material

Additional file 1 **Wells and springs original data set in Bq/L used to perform the related analysis**. The file shows the original calculations for the samples taken from each well and spring mentioned in the study and how the final calculations were derived. Click here for file

Additional file 2 **Residential tap water original data set in Bq/L used to perform the related analysis**. The file shows the original calculations for the samples taken from each residential tap water mentioned in the study and how the final calculations were derived. Click here for file

Additional file 3 **Old city original data set in Bq/L used to perform the related analysis**. The file shows the original calculations for the samples taken from each old city site mentioned in the study and how the final calculations were derived. Click here for file
